# Students’ acceptance and use of generative AI in pharmacy education: international cross-sectional survey based on the extended unified theory of acceptance and use of technology

**DOI:** 10.1007/s11096-025-01936-w

**Published:** 2025-06-04

**Authors:** Mohamed Hassan Elnaem, Betul Okuyan, Naeem Mubarak, Abrar K. Thabit, Merna Mahmoud AbouKhatwa, Diana Laila Ramatillah, AbdulMuminu Isah, Ali Azeez Al-Jumaili, Nor Ilyani Mohamed Nazar

**Affiliations:** 1 School of Pharmacy and Pharmaceutical Sciences, Ulster University, Coleraine, BT52 ISA UK; 2 Department of Clinical Pharmacy, Faculty of Pharmacy, Marmara University, Istanbul, Turkey; 3 Department of Pharmacy Practice, Lahore Medical and Dental College, University of Health Sciences, Lahore, Pakistan; 4 Pharmacy Practice Department, Faculty of Pharmacy, King Abdulaziz University, Jeddah, Saudi Arabia; 5 Department of Clinical Pharmacy and Pharmacy Practice, Faculty of Pharmacy, Alexandria University, Alexandria, 5372066 Egypt; 6 Faculty of Pharmacy, Universitas 17 Agustus 1945, Jakarta, Jakarta Indonesia; 7 Department of Clinical Pharmacy and Pharmacy Management, University of Nigeria, Nsukka, 410001 Enugu State Nigeria; 8 College of Pharmacy, University of Baghdad College of Pharmacy, Baghdad, Iraq; 9 Kulliyyah of Pharmacy, International Islamic University Malaysia (IIUM), Kuantan, Malaysia

**Keywords:** Generative AI, Pharmacy education, Pharmacy students, Technology acceptance, UTAUT framework

## Abstract

**Background:**

Generative artificial intelligence (GenAI) has significant potential implications for pharmacy education, but its ethical, practical, and pedagogical implications have not been fully explored.

**Aim:**

This international study evaluated pharmacy students’ acceptance and use of GenAI tools using the Extended Unified Theory of Acceptance and Use of Technology (UTAUT).

**Method:**

A cross-sectional survey of pharmacy students from nine countries during the first half of 2024 assessed GenAI usage patterns, curricular integration, and acceptance via the Extended UTAUT framework. After appropriate translation and cultural adaptation, exploratory factor analysis (EFA) identified key adoption factors.

**Results:**

A total of 2009 responses were received. ChatGPT and Quillbot were the tools most frequently utilised. EFA identified three key dimensions: Utility-Driven Adoption, Affordability and Habitual Integration, and Social Influence. Students rated performance and effort expectancy highly, highlighting their perceived usefulness and ease of use of GenAI tools. In contrast, habit and price value received lower ratings, indicating barriers to habitual use and affordability concerns. Gender disparities were noted, with males demonstrating significantly higher acceptance (*p* < 0.001). Additionally, country-specific differences were evident, as Malaysia reported a high performance expectancy, while Egypt exhibited low facilitating conditions. Over 20% indicated an over-reliance on GenAI for assignments, raising ethical concerns. Significant gaps were observed, such as limited ethical awareness—only 10% prioritised legal and ethical training—and uneven curricular integration, with 60% reporting no formal exposure to Generative AI.

**Conclusion:**

Findings reveal critical gaps in ethical guidance, equitable access, and structured GenAI integration in pharmacy education. A proactive, context-specific strategy is essential to align technological innovation with pedagogical integrity.

## Impact statements

- Pharmacy programs should include GenAI literacy modules that emphasise ethical use, critical evaluation of outputs, and responsible integration in clinical pharmacy learning.
- Affordability issues and uneven adoption rates necessitate subsidies or partnerships to ensure equitable access to GenAI tools, particularly in low—and middle-income countries.
- Establish clear institutional guidelines that consider the role of peer-led training programs, acknowledging the impact of social dynamics on GenAI adoption.

## Introduction

Higher education has recently witnessed major advances focused on integrating digital tools in teaching and learning [1]. Integrating generative artificial intelligence (GenAI) in pharmacy education is a growing area of interest that aligns with the broader trends in higher education. The rapid adoption of GenAI tools has been noted across various educational contexts, with students using these technologies for tasks ranging from idea generation to academic communication [2]. GenAI offers potential benefits such as simplifying complex concepts, creating study aids, and enhancing professional communication skills, which are crucial for mastering the pharmaceutical curriculum [3].

The availability of GenAI tools and applications has expanded opportunities for pharmacy students, but it has also raised concerns about ethical issues, and the accuracy and reliability of AI-generated content [4]. A Japanese study among pharmacy students highlighted the need for students to be educated on AI fundamentals to help them adopt these tools effectively [5]. Further, research among Nigerian pharmacy students identified knowledge gaps in relation to this technology, although positive perceptions towards its use, highlighting the need for further student-directed initiatives on the responsible GenAI academic uses [6]. Notably, the potential for over-reliance on these tools could impact students’ critical thinking and learning autonomy, necessitating careful consideration of how these technologies are implemented in educational settings [7]. Moreover, with a lack of proactive measures and guidance, there might be a risk that students will get used to using these tools as registered pharmacists without considering the impact on organisational requirements and patient safety [8]. Therefore, tailored training programs to equip students with key skills to evaluate and use GenAI are required to foster responsible integration [9].

While there is a well-defined policy governing the use of GenAI in some educational settings [10], educators need time to develop their GenAI skillsets [11]. Therefore, it is still questionable whether pharmacy students possess the essential GenAI literacy skills, making it imperative to conduct an assessment that comprehensively examines the dynamics of GenAI integration in academic activities. Investigating students’ perceptions of this rapidly evolving technology could help inform strategies that enhance the learning experience and guide further efforts on digital integration within pharmacy education [12, 13].

The unified theory of acceptance and use of technology (UTAUT) framework and its extension, which considers constructs such as performance expectancy, effort expectancy, social influence, and facilitating conditions, provides a robust lens to examine these dynamics [14, 15]. Through pharmacy education-oriented large-scale assessment, this research contributes to the ongoing discourse on the role of GenAI in education, offering insights that could inform policies and practices that support the effective integration of GenAI in pharmacy education.

### Aim

This international study aimed to evaluate pharmacy students’ acceptance and usage patterns on GenAI tools from nine countries using the Extended Unified Theory of Acceptance and Use of Technology (UTAUT) framework (including performance expectancy, effort expectancy, social influence, facilitating conditions, price value, habit and behavioural intentions).

### Ethics approval

The ethics research committees at Alexandria University, Egypt, and Marmara University, Türkiye, reviewed and approved the study protocol, followed by a few institutional ethical or administrative approvals from other participating institutions as needed. The introductory page of the online form included the participation information sheet and informed consent, which required approval before accessing the main survey. By approving the consent form, the participants were deemed to have consented to participate in this research. They were also free to withdraw their consent during the study. No compensation was provided to the participants.

## Method

### Study design

A cross-sectional online survey-based study was conducted among pharmacy students in 9 countries with relatively large pharmacy student populations in Asia, the Middle East, Africa, and Europe: Egypt, Türkiye, Indonesia, Pakistan, Iraq, Nigeria, Malaysia, Saudi Arabia, and the United Arab Emirates. The study used a validated, self-administered questionnaire prepared in English, Turkish, and Arabic on Google Forms. The study coinvestigators in each country disseminated all forms via private social media and other educational platforms, such as Microsoft Teams. Participants selected the language of the form and responded to only 1 version to avoid duplicate responses. The form settings were adjusted to limit only one response per participant. The data were collected in the first half of 2024, with slight variations in the duration at each study site.

### Inclusion and exclusion criteria

The study involved undergraduate and postgraduate pharmacy students who studied for at least one full semester at one of the involved institutions. Students from different pharmacy programs (for example, BPharm and PharmD) across all years of the study and training were eligible to participate. Students from different health education programmes and those on study leave were excluded.

### Sample size

This study was not primarily focused on cross-country comparisons but aimed to provide a comprehensive overview of the current integration of GenAI in pharmacy education. With an estimated proportion of 50% and a 95% confidence interval, we determined that a minimum of 77 to 120 students would be required in each institution, assuming at least one principal pharmacy school would participate [16, 17]. This minimum threshold was met successfully across seven out of nine participating institutions. The remaining two faced challenges due to smaller student populations and recent similar studies conducted nationwide. Consequently, they were not considered in the cross-country subsection of the study findings presentation.

### Instrument structure, translation, validity and pilot testing

The questionnaire was divided into four sections: demographic data, frequency of usage of GenAI tools, questions on the acceptance and use of GenAI tools in pharmacy education based on the Extended UTAUT Model, and items related to current usage and preferences for training and learning about GenAI tools. Given that all items based on the extended UTAUT model have been validated, a panel of five academics who are pharmacy practice experts evaluated the content validity of the final compiled four sections to ensure content relevance, coherence and alignment with study objectives. The content validity index was calculated for all items, ensuring it exceeded the minimum recommended level of 0.78. Translation, cultural adaptation, and pilot testing for face validity among pharmacy students were subsequently performed for all non-English instrument versions [18]. All model-based items have a given score on a scale from 1 to 5 across all different constructs. Finally, the total score per construct and individual item scores were calculated and compared accordingly.

### Statistical analysis

The current study used the IBMSPSS statistics for Windows, Version 29.0 (IBMCorp Released 2024. IBM SPSS Statistics for Windows, Version 29.0; IBMCorp) to analyse the data and Python to create visualisations. Descriptive statistics were employed in terms of frequencies and percentages. Each UTAUT dimension score was calculated on a 5-point Likert scale ranging from 1 (Strongly Disagree) to 5 (Strongly Agree). Overall construct scores and individual items were presented as medians and interquartile ranges because they violated the normal distribution. Nonparametric tests were used where appropriate to investigate whether the distribution of construct scores differs by demographics. A *P* value of < 0.05 was considered statistically significant for all other comparisons. Exploratory Factor Analysis (EFA) was conducted to examine the underlying structure of the initially developed 26-item scale using IBM SPSS Statistics 29. Principal Axis Factoring (PAF) was employed as the extraction method, with Promax oblique rotation applied to account for potential correlations among factors. The suitability of the data for factor analysis was confirmed via the Kaiser–Meyer–Olkin (KMO) measure of sampling adequacy and Bartlett’s test of sphericity. Factors were retained based on eigenvalues > 1 (Kaiser’s criterion), supported by scree plot inspection. The final factor solution was evaluated for theoretical coherence and reliability, and factor correlations were examined to assess relationships between constructs.

## Results

### Overall characteristics of the study participants

A total of 2009 responses were received. Most respondents are female (68.4%), while males constitute about 31.6%. Across nine countries representing different parts of the world, the highest participation was from Pakistan, Egypt, Indonesia and Malaysia. Table 1 displays general information about study participants.

**Table 1:** General information of study participants (N = 2009)

Characteristic	Frequency (N)	Percent (%)
Gender		
Male	634	31.6
Female	1375	68.4
Country of residence		
Iraq	263	13.1
Egypt	362	18.0
Pakistan	399	19.9
Indonesia	278	13.8
Nigeria	168	8.4
United Arab Emirates	26	1.3
Saudi Arabia	53	2.6
Malaysia	272	13.5
Turkiye	188	9.4
Level of Study		
1st year student	351	17.5
2nd-year student	436	21.7
3rd-year student	183	9.1
4th-year student	288	14.3
5th-year student	634	31.6
6th year (Intern)	53	2.6
PG-Masters/PhD	61	3.0
Unrecorded/unreported	3	0.1
Type of Educational Institute		
Governmental	1211	60.3
Private	798	39.7
Educational Program		
Bachelor’s degree	838	41.7
PharmD	1097	54.6
PG-MSc/PhD	74	3.7
Self-studying hours per week		
< 10 h	1047	52.1
10–20 h	616	30.7
21–30 h	245	12.2
31–40 h	60	3.0
> 40 h	41	2.0
Academic performance up to the previous semester/annual exam		
Excellent	411	20.5
Very good	615	30.6
Good	420	20.9
Average	203	10.1
Below average	100	5.0
Poor	20	1.0
Unrecorded/unreported	240	11.9

### Generative AI tools usage frequency

**Fig. 1:**
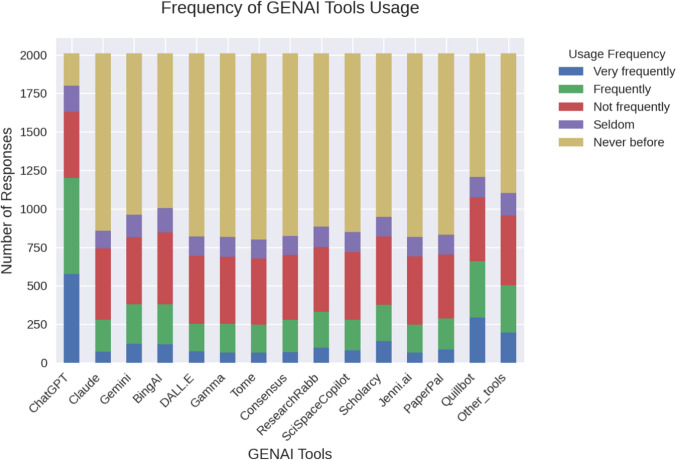
Frequencies of use of GenAI tools (n = 2009)

The most frequently used AI tools include ChatGPT and Quillbot, in addition to several other tools apart from the given choices, such as Poe AI, Canva AI, and Snapchat AI. Tools for specific academic uses with paid full user licences, such as Consensus, Gamma, and Tome, were less frequently used among study participants. Figure 1 shows approximate frequencies of using GenAI tools.

### Acceptance and use of generative AI tools: extended UTAUT-exploratory factor analysis

#### Factor assignment and loadings

Exploratory factor analysis (EFA) addressed the study’s exploratory aims, uncovered context-specific factor relationships, and refined the extended UTAUT model for GenAI in pharmacy education. Future research could build on these findings by validating the derived factor structure using CFA in targeted, less heterogeneous populations. EFA revealed a three-factor solution explaining 65.7% of the total variance derived from the Extraction Sums of Squared Loadings. The KMO value of 0.96 and significant Bartlett’s test (χ^2^ = 44,382.06, *p* < 0.001) confirm the appropriateness of factor analysis. The Promax rotation yielded a clear factor structure with factor loadings ranging from 0.42 to 0.94. The first factor explained 54.8% of the total variance, with subsequent factors 2 and 3 contributing progressively smaller proportions of variance at 7.2% and 3.7%. Two items (FC4 and PV1) were iteratively removed due to nonsignificant loadings with any of the factors (< 0.40). The final factor solution demonstrated strong construct validity, with each factor representing distinct yet interrelated psychological dimensions aligned closely with the original theoretical framework. The key factors identified by EFA and their relevant constructs were as follows:

Factor 1. Utility-Driven Adoption. Constructs Included: Performance Expectancy (PE), Effort Expectancy (EE), Hedonic Motivation (HM), Facilitating Conditions (FC). This factor represents students’ perception of GenAI tools as practically beneficial (PE), easy to learn and use (EE), enjoyable (HM), and supported by adequate institutional resources (FC).

Factor 2. Affordability and Habitual Integration. Constructs Included: Price Value (PV), Habit (HT), Behavioral Intention (BI). This factor captures students’ cost–benefit evaluations (PV) of GenAI tools, their routine reliance on these tools (HT), and their planned future use and sustained engagement (BI).

Factor 3: Social Influence. With only one construct included, this factor reflects the role of peers, mentors, and societal pressures in shaping students’ decisions to adopt GenAI tools.

#### Constructs correlation

**Fig. 2:**
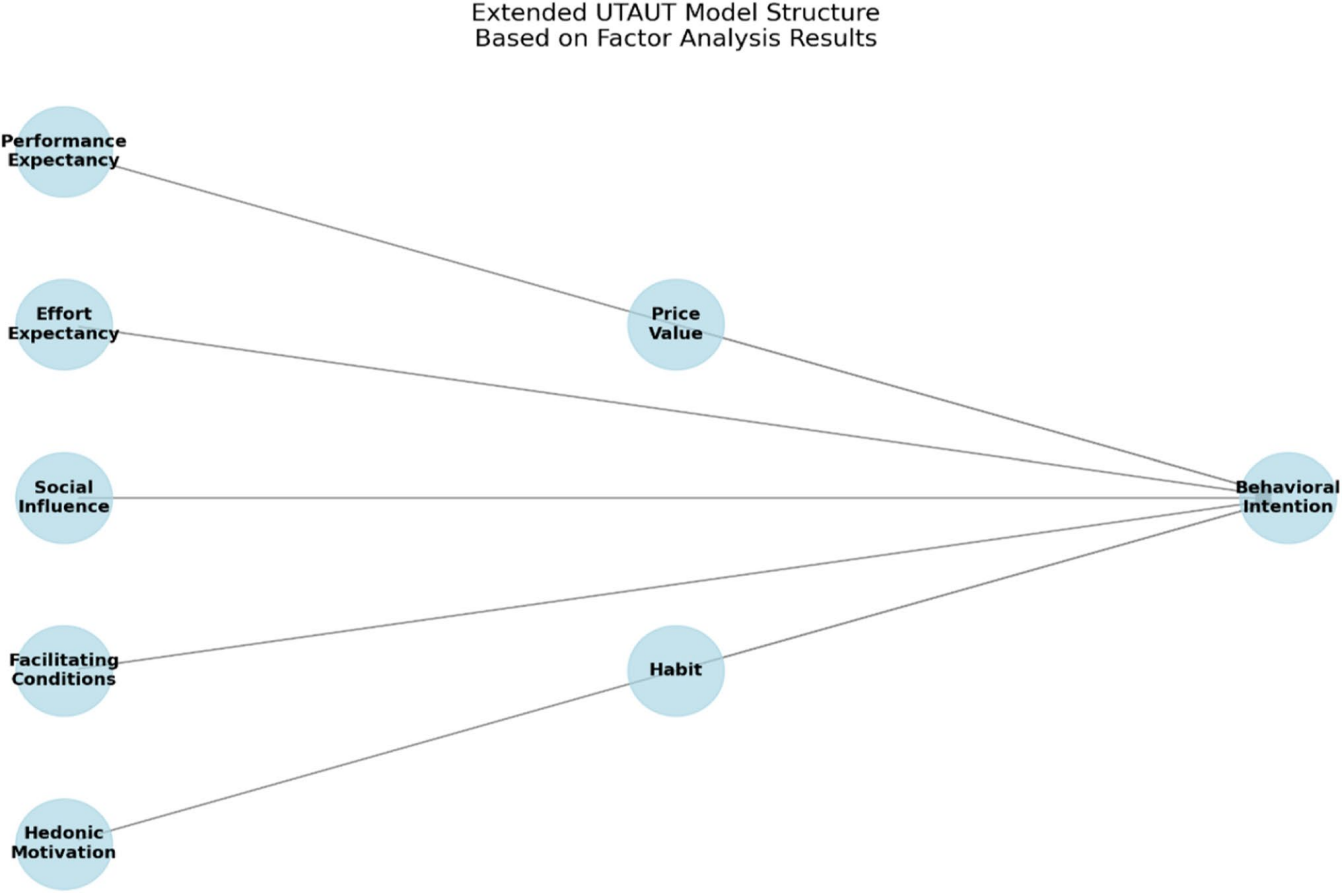
Overall extended UTAUT model structure based on the factor analysis results

The constructs correlation matrix indicated moderate to strong relationships where the factors represent related yet distinct constructs. Since factors are allowed to correlate (Promax rotation), this correlation reflects an overlap in the constructs. All constructs show significant relationships with Behavioral Intention (BI), particularly Performance Expectancy (PE), Hedonic Motivation (HM), and Habit (HT). Finally, overall Cronbach’s alpha reliability scores were calculated, confirming strong internal consistency for all constructs (PE: 0.94, EE: 0.95, SI: 0.95, FC: 0.94, HM: 0.97, PV: 0.91, HT: 0.94, BI: 0.95). Figure 2 shows the overall extended UTAUT model structure based on the factor analysis results.

### Acceptance and use of generative AI tools extended UTAUT-construct items

The extended UTAUT model encompasses eight different constructs with a total of 24 items as per the EFA findings. Every construct has two to four individual items assessed on a score scale ranging from 1 (strongly disagree) to 5 (strongly agree). A total score for every construct is presented to inform areas of the highest agreement and those that need further consideration. The median scores for the constructs present the following insights: Performance Expectancy (PE) has a median of 4 and an interquartile range (IQR) of 2, reflecting high expectations accompanied by notable variability in responses. Effort Expectancy (EE), also with a median of 4, boasts a narrower IQR of 1.5, indicating a strong belief in ease of use with relatively consistent feedback. Social Influence (SI) scores a median of 3 and an IQR of 1, suggesting a neutral to positive impact of social factors. Habit shows the lowest median score along with the widest IQR, highlighting a moderate level of habit formation coupled with considerable variation in responses. Price Value stands at 3.5 with an IQR of 1, indicating a moderately positive perception of value. Finally, behavioural intention (BI) is recorded at 4 with an IQR of 1, which indicates a firm intention to use GENAI tools consistently among respondents. The findings indicate that pharmacy students tend to have favourable perceptions of GENAI tools, especially regarding their performance, ease of use, and intention to use them. However, habit formation reflects more moderate levels, suggesting that while students recognise the value of these tools, consistent usage patterns are still developing. Table 2 presents all individual and total scores for all constructs of the extended UTAUT model.

**Table 2:** Acceptance and use of generative AI tools expressed in percentages of responses to items of the Extended UTAUT Model constructs (N = 2009)

	Strongly disagree %	Disagree %	Neutral %	Agree %	Strongly agree %	Construct score Median (IQR)
PE1 [I find generative AI tools useful in my daily student life.]	1.7	3.5	22.5	38.4	33.8	4 (2)
PE2 [Using generative AI tools helps me accomplish academic tasks more quickly.]	2.3	3.3	22.3	40.9	31.2	
PE3 [Using generative AI tools increases my academic productivity.]	2.9	6.3	28.0	36.5	26.3	
EE1 [Learning how to use generative AI tools is easy for me.]	2.3	5.5	25.7	39.0	27.6	4 (1.5)
EE2 [My interaction with generative AI tools is clear and understandable.]	2.4	5.7	27.7	38.2	26.0	
EE3 [I find generative AI tools easy to use.]	2.1	4.6	24.0	41.4	27.8	
EE4 [It is easy for me to become skillful at using generative AI tools.]	2.4	6.4	29.1	37.7	24.4	
SI1 [People who are important to me think that I should use generative AI tools.]	3.6	9.8	37.2	31.7	17.6	4 (1)
SI2 [People who influence my behavior think that I should use generative AI tools.]	4.0	11.5	36.8	31.4	16.3	
SI3 [People whose opinions that I value prefer that I use generative AI tools.]	3.5	10.7	37.1	32.6	16.1	
HM1 [Using generative AI tools is fun.]	2.4	4.2	25.2	41.5	26.7	4 (2)
HM2 [Using generative AI tools is enjoyable.]	2.2	4.1	25.2	40.9	27.6	
HM3 [Using generative AI tools is very entertaining.]	2.2	4.9	28.4	37.9	26.6	
HT1 [The use of generative AI tools has become a habit for me.]	9.6	14.4	32.0	27.2	16.8	3 (2)
HT2 [I am addicted to using generative AI tools.]	14.4	16.7	32.9	23.0	12.9	
HT3 [I must use generative AI tools.]	10.6	13.8	32.9	27.6	15.1	
PV2 [Generative AI tools provide good value for money.]	3.3	8.0	38.1	32.2	18.5	3.5 (1)
PV3 [I can afford the price of all essential generative AI tools that provide value to my academic work.]	9.1	14.4	34.8	26.9	14.8	
FC1 [I have the resources necessary to use generative AI tools.]	2.7	7.8	30.7	36.8	22.1	4 (1)
FC2 [I have the knowledge necessary to use generative AI tools.]	2.7	6.7	28.8	39.7	22.1	
FC3 [Use of generative AI tools is compatible with other technologies I use.]	1.8	5.0	28.7	41.8	22.6	
BI1 [I intend to continue using generative AI tools in the future.]	3.1	5.4	30.4	38.7	22.5	4 (1)
BI2 [I will always try to use generative AI tools in my daily student life.]	4.3	9.8	34.1	32.8	19.0	
BI3 [I plan to continue to use generative AI tools frequently.]	3.3	8.5	32.6	33.9	21.7	

All metrics are on a 5-point Likert scale where 1 = Strongly Disagree and 5 = Strongly Agree

^*^UTAUT: Unified theory of acceptance and use of technology

^*^Performance Expectancy (PE), Effort Expectancy (EE), Hedonic Motivation (HM), and Facilitating Conditions (FC), Price Value (PV), Habit (HT), Behavioural Intention (BI), Social Influence (SI)

### Trends in UTAUT construct overall scores across countries

**Fig. 3:**
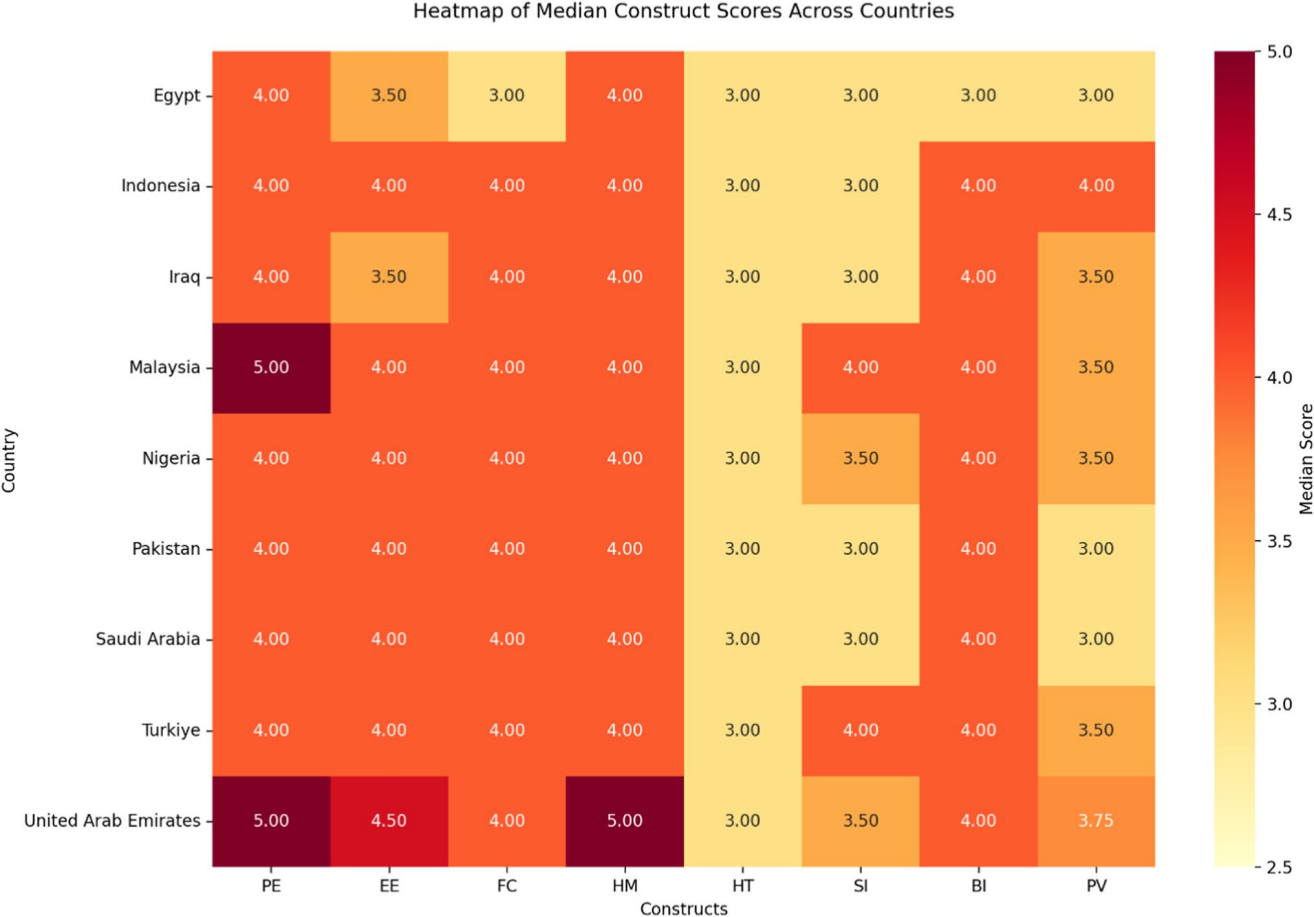
Heatmap of median scores of UTAUT constructs across countries

Although not the primary objective of this study, comparing overall construct scores across countries was necessary to help set priority areas for advocating responsible GenAI use. Excluding the countries with relatively smaller sample sizes, such as UAE and Saudi Arabia, the rest of the comparisons across different constructs reveal that Malaysia has been ranked highest in performance expectancy. Egypt and Iraq ranked the lowest for the construct of effort expectancy. Egypt had the lowest median across all countries for facilitating conditions and behavioural intention to use GenAI tools. Türkiye and Malaysia had the highest median for the construct of social influence, followed by Nigeria. The lowest medians for price values were reported in Pakistan and Egypt, while the highest was for Indonesia. All countries consistently shared the same median for the habit construct, reflecting a general trend. Investigating the distribution of median construct scores across countries showed significant differences across all construct domains, underpinning diverse underlying conditions of acceptance and use of GenAI among pharmacy students in different countries. Figure 3 shows the overall construct scores of acceptance and use of GenAI across various countries.

### Key tasks, curricular events and learning interests related to GenAI tools

**Fig. 4:**
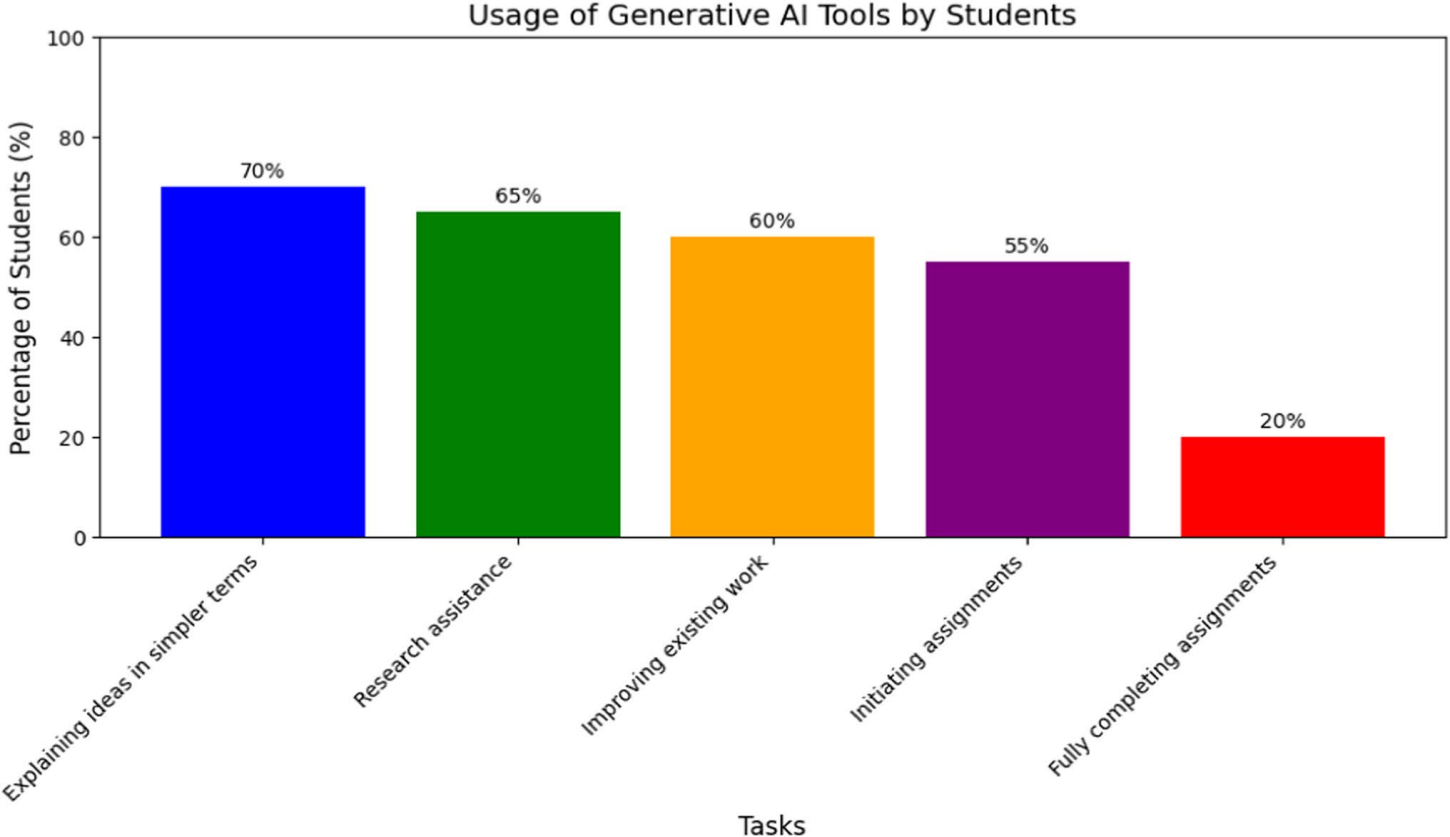
Patterns of use of generative AI tools by pharmacy students

Concerning the top tasks for which students use GenAI tools, the most common use was for explaining ideas in simpler terms, with approximately 70% of students adopting GenAI tools for this purpose. This was followed by research assistance use, where about 65% of students use AI tools to help with research and find facts, quotes, or resources. Around 60% of students used GenAI tools to improve existing work and enhance their previous work. Approximately 55% of students used GenAI tools to initiate their assignments or generate initial ideas. Finally, about 20% reported relying on GenAI tools to fully complete assignments. While this is a significant percentage, it highlights areas for potential improvement in the responsible use of these tools in academic tasks. Figure 4 illustrates key academic uses of GenAI among pharmacy students. Regarding the load of formal GenAI-related events embedded into the curricula, approximately 60% of participants reported no exposure, and the rest reported minimal exposure through their studies. By investigating learning needs about GenAI in the pharmacy curriculum, approximately 45% reported needs related to practical skills with these tools, while only 10% highlighted needs related to legal and ethical aspects. About 20% and 17% still need support on GenAI theory background and future perspectives, respectively.

### Associations between demographics and median scores of UTATUT constructs

Our analysis revealed significant gender differences across all UTAUT constructs, with male students generally showing higher acceptance levels (*p* < 0.001). Investigating the distribution of median construct scores across different levels of study showed significant differences across all construct domains (all *p* < 0.001) except for social influence (*p* = 0.099), with third-year students demonstrating the highest performance expectancy and effort expectancy. Furthermore, academic performance showed limited impact, with significant differences only in Effort Expectancy (*p* = 0.025) and Facilitating Conditions (*p* = 0.011). Interestingly, students with higher academic performance, such as excellent (adjusted *p* = 0.037) and very good (adjusted *p* = 0.031), reported higher median scores than average students. These findings suggest that GenAI tool acceptance in pharmacy education is more strongly influenced by gender and educational level than academic performance, with male students and those in their mid-study years showing generally higher acceptance levels.

## Discussion

### Statement of key findings

This international study on pharmacy students’ perspectives regarding GenAI comprehensively examines technology adoption across nine countries, utilising the Extended UTAUT framework. The research included a cross-sectional survey targeting pharmacy students in various geographical regions, specifically Asia, the Middle East, Africa, and Europe, encompassing Egypt, Türkiye, Indonesia, Pakistan, Iraq, Nigeria, Malaysia, Saudi Arabia, and the United Arab Emirates. This study represents one of the largest investigations focused on accepting and using GenAI among pharmacy students. Employing the extended UTAUT framework delivers an in-depth analysis of the acceptance and usage domains based on eight key constructs of the model. This thorough assessment across different contexts aims to guide future initiatives and strategies for fostering responsible GenAI use among pharmacy students.

### Strengths and weaknesses

Through a theory-driven large-scale assessment, this study addresses an important topic, providing detailed findings on the acceptance and use of GenAI globally and highlighting differences across UTAUT domains based on country, gender, level of study and academic performance. This may help enhance the understanding of GenAI adoption in pharmacy education. However, this study has several limitations, including its cross-sectional design that limits causal inference, potential selection bias in participant recruitment, geographical concentration in specific regions, and self-reported data that might introduce response bias. Finally, the analysis of the associated factors with GenAI was informative but not extensive enough to uncover all potential factors that might impact adoption. These factors necessitate a careful interpretation and suggest the need for future research to thoroughly examine GenAI’s evolving role in pharmacy education.

### Interpretation

The research uncovered several significant findings regarding using GenAI among pharmacy students. Expectedly, the patterns of frequency and preferred tools highlighted commonly used resources such as ChatGPT, followed by academic tools for content writing like Quillbot. This aligns with previous research that underpinned these academic writing tools, which are widely common in higher education settings [11, 19]. In contrast, specific single-purpose academic tools like Gamma and Tome experienced minimal adoption. Additionally, many respondents mentioned other emerging tools beyond the commonly referenced GenAI options. Earlier research highlighted that the choices between different tools are continuously changing and impacted by perceived efficiency, interaction, and intention [20], making multi-purpose tools appealing options to satisfy several needs through one platform. The interest in emerging GenAI tools indicates that educational institutions should enhance awareness and provide continuously updated guidance on these resources.

The research provided valuable insights into how pharmacy students engage with various GenAI tools across academic tasks. The wide array of applications for GenAI tools demonstrates their adaptability and integration into multiple facets of academic work. The high percentages of students utilising these tools for explanation, research, and improvement indicate that they primarily view them as supportive resources to enhance their learning and output quality. This wide range of uses is impacted by opportunities offered by GenAI to streamline learning, research, and assessment processes while making it a personalised and engaging experience [21]. The comparatively lower percentage of students relying on GenAI to complete assignments entirely is a positive sign, suggesting that most students are not overly dependent on these tools. However, this also highlights the need to reinforce the ethical aspects of interacting with these tools and areas where additional support or guidelines may be essential [22]. This data mainly benefits educators and institutions, highlighting the need to effectively integrate GenAI tools into the curriculum and guide students in their usage.

The analysis of the Extended UTAUT model revealed insightful findings at the construct level. Performance and effort expectancy were among the top-scoring constructs, indicating that students primarily perceive generative AI tools as valuable, accessible, and efficient for completing academic tasks. Previous studies highlighted that performance expectancy, effort expectancy, and social influence significantly influence the intention to use GenAI, but only performance expectancy and social influence directly impact academic performance [23]. Conversely, habit followed by price value constructs received the lowest scores, suggesting that adopting AI tools has not yet become a deeply established practice among students, while affordability continues to be a significant concern for individual users. This may raise the concern of equitable access to these tools, which requires a clear organisational perspective on a structured and targeted strategy to integrate GenAI [24].

The exploratory factor analysis (EFA) revealed a three-factor solution that partially aligns with the original UTAUT framework while introducing novel interactions among constructs. Most notably, Habit (HT) and Price Value (PV) loaded together under Factor 2 (Affordability and Habitual Integration). The coupling of *Habit* and *Price Value* suggests that students’ habitual use of GenAI tools is closely tied to affordability barriers, particularly in lower-income regions. For instance, tools requiring paid licenses were underutilised (Fig. 1). Research indicates that technology adoption in developing economies is significantly influenced by cost-effectiveness, even during the initial stages of habitual usage [25]. In such contexts, habitual use may only emerge if tools are perceived as financially accessible, creating a feedback loop where affordability reinforces routine engagement [26].

Moreover, the standalone Social Influence (SI) factor (Factor 3) aligns with theoretical emphasis on peer and mentor pressures. Existing interpretations of social influence in technology adoption suggest that while social influence may align with UTAUT’s emphasis on peer pressures, its independence from constructs like Performance Expectancy could vary across cultures [27]. In Southeast Asian societies, social norms significantly impact technology adoption. In contrast, social influence may be less tied to utility-driven adoption in contexts with weaker institutional support. These findings highlight the necessity of contextualising UTAUT extensions for emerging technologies such as GenAI. Our research shows that cost and cultural factors can influence theoretical relationships, especially in diverse, cross-national samples.

Curriculum-related findings revealed significant gaps in formal GenAI education. An overwhelming 60% of participants reported no exposure to GenAI-related events within their pharmacy curriculum. Merely 10% highlighted legal and ethical considerations. This disparity highlights the need for comprehensive GenAI integration strategies in pharmacy education. A relatively small-scale international study recruited 387 pharmacy students and highlighted a positive attitude towards this technology, indicating a need for relevant education and training [28]. This raises important questions about whether pharmacy educators are equipped to lead by example and upskill students’ skills in this area. Beyond the broad applications of GenAI use for generating study aids, brainstorming ideas, and offering practice opportunities for clinical problems, educators have started to develop successful examples of integrating this technology into the pharmacy curriculum in a subject-specific manner [29, 30].

Compared to previous work conducted among pharmacy students, the current study highlighted country-specific variations in extended UTAUT constructs and provided additional depth to the analysis. Malaysia had the highest ranking in performance expectancy, while Egypt and Iraq had the lowest scores for effort expectancy. Egypt ranked lowest in facilitating conditions and behavioural intention to use GenAI tools. Türkiye and Malaysia scored highest in social influence. Pakistan and Egypt recorded the lowest price values, while Indonesia had the highest. These variations highlight the complex landscape of GenAI acceptance across different educational and cultural contexts, informing the need for a context-specific approach to promoting responsible GenAI integration in pharmacy education [23].

Finally, our analysis revealed notable gender differences in the UTAUT constructs, with male students demonstrating higher acceptance. This is consistent with an earlier study that showed better perceptions and a higher pattern of use for broader applications among males compared to females, who were more specific and critically evaluating the usefulness of adopting these tools [31]. On the other hand, a recent study based on the technology acceptance model reported no significant gender-based differences in the perceived effectiveness of GenAI writing tools [32]. In a small study among second- and third-year US pharmacy students to investigate perceptions on utilising ChatGPT for clinical presentations, third-year students were more familiar and confident [33], consistent with our data that showed that third-year students exhibited the highest performance and effort expectancy. In the present study, academic performance was found to influence only Effort Expectancy and Facilitating Conditions, while higher-achieving students reported superior median scores compared to their average peers. Previous studies have shown mixed results regarding the relationship between academic performance and attitudes toward adopting technology. Some research indicates that students with higher academic achievement tend to have a more positive attitude toward technology adoption [34], while other studies have not found a significant impact of academic performance on this attitude [35]. The findings suggest that acceptance of GENAI tools in pharmacy education may be influenced more significantly by demographic factors, such as gender and educational level, than by academic performance. This highlights the importance of considering these factors in the development of future initiatives.

### Further research

This study reveals implications for GenAI in pharmacy education. Three policy priorities are identified for responsible GenAI utilisation. First, establishing clear ethical standards and policies is crucial to maintaining academic integrity while maximising GenAI’s potential [36]. Comprehensive ethical guidelines must be developed to mitigate concerns regarding excessive reliance on GenAI for academic tasks [22]. Such guidelines are vital for preserving academic integrity and enhancing critical thinking skills. Second, curriculum Integration is essential to incorporate mandatory GenAI literacy modules in pharmacy programs, focusing on ethical usage and skill development (e.g., critical evaluation of AI outputs) while exploring future directions. Building capacity among pharmacy educators and developing structured strategies for GenAI integration into the curriculum is imperative to uphold quality standards and improve efficiency [37]. Third, context-specific Training that should utilise cultural strengths and social influences through peer-led training initiatives while ensuring equitable access to specific GenAI tools via institution-sponsored programs.

## Conclusion

This international study explored pharmacy students’ perspectives on the acceptance and use of GenAI tools, revealing significant gaps in ethical awareness, equitable access, and structured integration. The findings highlight the need for a proactive and strategic approach to integrating these tools, emphasising the importance of tailoring solutions to specific contexts while maintaining a balance between technological innovation and pedagogical integrity.
